# Stem cell decisions: A twist of fate or a niche market?

**DOI:** 10.1016/j.semcdb.2014.02.014

**Published:** 2014-10

**Authors:** Jens Januschke, Inke Näthke

**Affiliations:** Cell and Developmental Biology, College of Life Sciences, University of Dundee, Dundee DD1 5EH, UK

**Keywords:** Spindle orientation, Centrosome segregation, Stem cells, Asymmetric cell division

## Abstract

Establishing and maintaining cell fate in the right place at the right time is a key requirement for normal tissue maintenance. Stem cells are at the core of this process. Understanding how stem cells balance self-renewal and production of differentiating cells is key for understanding the defects that underpin many diseases. Both, external cues from the environment and cell intrinsic mechanisms can control the outcome of stem cell division. The role of the orientation of stem cell division has emerged as an important mechanism for specifying cell fate decisions. Although, the alignment of cell divisions can dependent on spatial cues from the environment, maintaining stemness is not always linked to positioning of stem cells in a particular microenvironment or `niche'. Alternate mechanisms that could contribute to cellular memory include differential segregation of centrosomes in asymmetrically dividing cells.

## Highlights

• Extrinsic and intrinsic cues that impact on stem cell biology.
• The importance to establish methods that allow to compare spindle orientation measurements.
• Mechanisms of centrosome segregation in asymmetrically dividing cells.

## 1 Introduction

One of the central questions in cell and developmental biology is how differences in cells are established and maintained. In multicellular organisms this problem is not restricted to development but is also relevant during tissue homeostasis in the adult. One mechanism for establishing different cell fates is asymmetric cell division. In this context, the transmission of cell fate information can occur through cell–cell communication, it can be established *via* intracellular polarity or it can be inherited from one cell generation to the next [1]. Stem cells are one cell type that can divide asymmetrically to produce a self-renewed stem cell and a daughter cell that will differentiate. Stem cells can also divide symmetrically to expand the stem cell pool. Increasing stem cell numbers or generating differentiating cells is a key process in building and maintaining tissues. In the context of stem cells the orientation of the mitotic spindle can influence the fate of daughter cells [1], [2]. The correct alignment of mitotic spindles is not only important in development but defects in this process are also associated with disease [3], [4]. It is thus not surprising that controlling the orientation of mitosis is an important issue for tissue morphogenesis [5], [6], [7]. The different requirements and contexts in which stem cells are found predict that a plethora of regulatory mechanisms operate to govern spindle orientation and cell fate decisions. Here we discuss intrinsic and extrinsic cues that are involved in asymmetric stem cell division and focus specifically on the contribution of selective centrosome segregation.

### 1.1 Principle concepts of spindle orientation

Invertebrate model systems have proven extremely useful for unraveling the general principles that underpin spindle orientation during asymmetric cell division. The genetic approaches possible in these model systems permit asking detailed questions about this process. They also enable identification and easy access of the cells under investigation. Importantly, most of the molecular principles of asymmetric division identified in *Drosophila* and *Caenorhabditis elegans* are highly conserved [1], [8], [9].

**Fig. 1:**
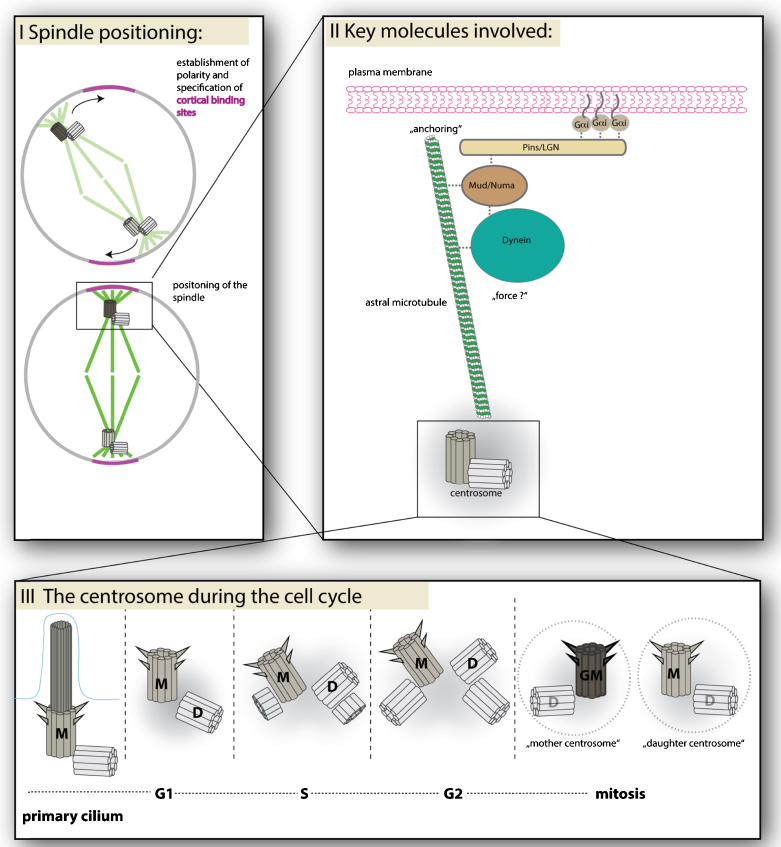
(I) Spindle orientation can involve establishment of localized domains at the cell cortex that can anchor astral microtubules. In some cases, these domains are established by proteins of the Par complex. Position of these domains can be specified through extrinsic as well as intrinsic signals. Once astral microtubules interact with these anchoring domains torque is exerted on the spindle causing it to rotate toward them. (II) The core components involved in many spindle positioning events are Galphai, Pins/LGN, Mud/Numa and Dynein. Myristylation of Galphai links it to the plasma membrane. Galphai can bind Pins/LGN and regulates the affinity of Pins for Mud. Mud can directly bind to microtubules but also cytoplasmic Dynein. Dynein is believed to provide at least part of the forces required to orient the spindle. (III) The centrosome is found at different configurations during the cell cycle and also provides asymmetry to the spindle since the centrosomes at each spindle pole can be distinguished by the age of the set of centrioles they carry. On the spindle one centrosome, the mother centrosome, contains the older set of centrioles. Centrioles typically duplicate during G1/S phase when a new centriole forms in the vicinity of each old centriole. M: mother centriole, D: daughter centriole, GM: Grandmother centriole (to indicate that one of the two centrioles that qualify as mother centrioles has formed a cell cycle earlier).

How is spindle orientation achieved? A series of events cooperate to position the spindle. In many instances two key events are required that are tightly coupled (Fig. 1). First, cell polarity needs to be established specifying cortical regions that can capture the spindle. Second, the spindle apparatus needs to be able to interact with the cortex. Typically, astral microtubules nucleated by centrosomes at the spindle poles serve this purpose. Common to this process in various contexts, is the contribution of a conserved, sophisticated molecular machinery that includes cortical and microtubule binding proteins in addition to molecular motors that can exert torque on the spindle. Our understanding of the key molecules involved in this machinery is steadily increasing [10].

In Brief, G alphai, LGN (ASG3 in *C. elegans* and Pins in *Drosophila*) and Numa (Lin-5 in *C. elegans*, Mud in *Drosophila*) constitute the conserved core set of molecules involved in spindle positioning (Fig. 1). G alphai can be myristoylated and binds to the cortex [11]. G alphai also regulates the activity of Pins by increasing its affinity for Mud [12]. Pins/LGN binds Mud/Numa [2], [13], [14], [15]. In turn, Numa/Mud can interact with cytoplasmic Dynein [16], [17], which can exert forces to orient the spindle. Hence, this protein complex can function in anchoring and positioning the spindle. These molecules also play important roles in directing spindle orientation in progenitor cells in the mouse neocortex, the chicken neural tube, and during symmetric divisions in developing epithelia [18], [19], [20], [21], [22]. The proteins involved seem to function similarly in different contexts. Nonetheless, how the orientation of mitotic spindles influences the outcome of progenitor/stem cell division varies and is not understood in many progenitor cells [23]. Another difficulty is that measuring spindle orientation reliably in complex stratified vertebrate tissues is more complex than in the simpler tissue structures of *Drosophila* or *C. elegans*.

### 1.2 Spindle orientation – how to measure it properly?

In vertebrates, the orientation of mitotic spindles is commonly used to classify symmetric and asymmetric divisions [24], [25], [26], [27]. Although the position of daughter cells does not necessarily predict the fate of resulting daughter cells, the alignment of mitotic spindles perpendicular to the tissue layer in which the mother resides, usually this corresponds to the apical surface, is considered asymmetric because the daughter cells inherit different proportions of apical polarity markers. The problem that arises especially in morphologically complex tissues is: what is used as reference to determine the orientation of the spindle? It is important to note that the methods used to measure mitotic spindle alignment have never been compared directly and the reference points used to report the angle of spindle orientation differ between investigators and systems [24], [25], [26], [27]. This may explain discrepancies between observations in the same system [24], [25], [26], [27]. In tissue that is curved like the base of the intestinal crypt, it becomes even more difficult to define relevant reference points or axes that relate to cell or tissue organization and more robust methods for these measurements in three-dimensional tissue are needed.

### 1.3 Stem cell compartment, plasticity and the niche concept

Additional complexity is added by the emerging view that at least some stem cell compartments have a high degree of plasticity. Within some tissues, several cell populations can act as stem cells in a context dependent manner. Which stem cell pool is the active one under a given set of circumstances? This important for understanding the role of spindle orientation in cell fate decisions and is particularly relevant in the stem cell compartment of the mouse intestine. In recent years much progress in understanding the biology of the stem cells at the base of intestine has been made revealing a high level of plasticity within this compartment [28].

Leucine-rich repeat containing G protein-coupled receptor 5 (LGR5) was identified as a marker of cells that can generate all the lineages normally present in the intestinal epithelium [29]. Within the epithelium, Paneth cells are secretory cells that are usually restricted to the crypt base where the antimicrobial peptides they secrete are thought to protect neighboring stem cells [30]. Previously, cells that reside at position +4, above the last Paneth cell, were identified as stem cells based on their ability to retain labeled DNA [31]. These so called +4 cells express low levels of LGR5 in addition to the marker Bmi1. Importantly, +4 cells can restore LGR5^Hi^ cells upon their depletion [32]. Similarly when +4 cells are specifically depleted, they are restored from the LGR5^Hi^ pool [33]. To complicate the situation further, a subset of Paneth cells can act as reserve stem cell pool when called upon in response to injury or disease [34]. Together these and other similar observations illustrate the high degree of plasticity that exists in this tissue between different pools of progenitor cells in this tissue. The high turn over of cells in the intestine makes it vital to maintain a constant supply of replacement cells. A highly dynamic stem cell compartment that includes back-up provisions ensures the survival of the organism. The molecular mechanisms that control these decisions remain a mystery but they are likely to include a complex interplay between different signaling pathways, differential adhesion between cells and basement membrane, and mechanical forces that act at the level of cells and tissue.

Stem cells usually reside in a particular environment called the niche, that hosts and maintains stem cells [35], [36]. One idea that has gained popularity is that the niche is the dominant factor in controlling stem cell fate by providing short-range signals that confer stemness on cells within their range. In the *Drosophila* germline, niche signals can even promote reversion of cells that are partially differentiated to become stem cells again [37], [38]. However, such powerful effects of the niche are not universal. In the case of the hair follicle, cells do not revert to a stem cell fate when they return to the niche after exiting and differentiating even when the niche is depleted of endogenous stem cells [39]. On the other hand, hematopoietic stem cells can leave the niche without loosing their stemness [40] and neural stem cells can exist and symmetrically self-renew outside their complex microenvironment [41].

In the case of the crypts in the intestine, Paneth cells secret important stem cell maintenance factors including Wnt [42]. If Paneth cells are experimentally ablated, however, stem cells are maintained *in vivo* [43]. Hence crypt stem cells have the capacity to compensate for the loss of Paneth cells and maintain stemness by other means. Similarly, murine neuroepithelial progenitor cells removed from their normal location produce neurons at normal frequency suggesting that their self-renewal capacity does not immediately rely on environmental signals [19]. Thus, mechanisms that are independent of a particular microenvironment can drive differentiation or stem cell self-renewal in some stem cell populations. This in turn suggests that at least some stem cells have the capacity to control self-renewal intrinsically or to self-organize a favorable environment to help them do so. Indeed, neural stem cells in the olfactory epithelium together with neighboring cells release factors that can negatively regulate self-renewal and proliferation to maintain homeostasis [36], [44]. Likewise epidermal stem cells can be the source of their own self-renewing signals as well as for the differentiating signals for their progeny [45].

These data question the universal validity of the classical concept that the niche provides all the cues required for normal stem cell maintenance and emphasize the need to consider additional mechanisms that can confer cell fate.

An emerging concept that can explain how cellular states are maintained between different generations proposes that cellular memory can be passed on from one cell to the next during division [46]. Prominent examples for mechanisms that could transmit information from one cell generation to the next include epigenetic modification of the chromosomes [47], the inheritance of the midbody, which can impact dramatically on cellular physiology and cell-fate determination [48], and asymmetric segregation of centrosomes and cilia [49]. These elements may provide the molecular basis for transmitting differential cell fate information. In the following sections we discuss what is known about such mechanisms in asymmetrically dividing cells, specifically stem cells, focusing on recent advances in understanding the mechanism and function of non-random centrosome segregation.

### 1.4 Asymmetric inheritance of centrosomes

Cell fate information could be carried directly by the spindle. Consistent with this idea, various organelles and mRNAs associate with the spindle to provide potential fate determinants to one or both daughter cells [50]. In this context, the centrosome is particularly important. Centrosomes segregate to the opposing poles of mitotic spindles each time a cell divides making them ideal vehicles for carrying information from one cell to another during division.

Centrosomes also provide a means to establish polarity in a spindle because they are intrinsically different, due to their duplication cycle [51]. At the core of a typical centrosome are two centrioles. Before new centrioles are produced, the two centrioles already present separate and each one acts as the site for the assembly of a new centriole. As a result, centrioles within each centrosome can be distinguished by age-reflected in the language used to describe the older centriole as “mother” and the younger centriole as “daughter”. Hence the ‘*mother centrosome’* carries the oldest set of centrioles whereas the ‘*daughter centrosome’* carries the younger set (Fig. 1).

Differences in the maturation of mother or daughter centrioles are reflected by structural differences and the unequal distribution of proteins [52]. Consequently, molecular differences exist between centrosomes that cells could use to distinguish between them.

Indeed, differential segregation of mother and daughter centrosomes has been observed in cells that divide asymmetrically. However, the direction of centrosome segregation is not always the same. In *Drosophila* male germ line stem cells [53] and in progenitor cells of the neocortex in mice [54] the mother centrosome stays within the stem cell in asymmetric divisions.

In budding yeast, where the phenomenon of differential centrosome segregation was first discovered [55] and in *Drosophila* larval neuroblasts [56], [57] the mother centrosome (or spindle pole body (SPB) in the case of yeast) leaves the old cell (the self-renewed stem cell in the case of neuroblasts) and segregates to the new daughter cells. This direction of segregation was also observed in cells from a neuroblastoma cell line where the daughter centrosome is inherited by the cell with progenitor potential [58].

### 1.5 Contribution of structural differences in centrosomes to biased centrosome segregation

The nature of centriole duplication causes the presence of centrioles with different states of maturity within a cell. Intriguingly, in system that display biased centrosome segregation like budding yeast, the Drosophila male germ line and Drosophila neuroblasts, the centrosomes (SPBs in the case of yeast) differ in their ability to nucleate microtubules during interphase [53], [56], [57], [59], [60], [61]. This could suggest that centrosome segregation patterns may be driven by differences in the ability to nucleate astral microtubules caused by structural variations that result from the maturation state of daughter *versus* mother centrioles.

In vertebrate cells mother and daughter centrioles vary in their ability to recruit components for microtubule nucleation in interphase [62]. This might be because centrioles require ∼1.5 cell cycles to fully mature to become a mother centriole. The maturation is accompanied by the formation of different types of appendages that may be involved in anchoring microtubules [63], [64]. Hence, the increased ability of the mother centriole to nucleate and/or anchor microtubules might confer an advantage for engaging with the microtubule binding sites at the cortex, which in turn enhances the probability of the mother centriole to be retained there. Although appendages do not form on mother centrioles in *Drosophila* [65], the mother centrosome of male germ line stem cells can nucleate a significant number of microtubules during interphase [53]. To ensure asymmetry of the process, such astral microtubules might then be captured by asymmetrically localized microtubule stabilizing proteins like the adenomatous polyposis coli protein (APC), which is restricted to the stem cell/hub cell interface [66].

Differences in the maturation of the SPB might also drive biased SPB segregation in budding yeast. The old SPB is guided into the bud and this requires the Kar9 protein, a protein with some sequence similarity to APC [67]. Importantly, the old SPB has the ability to nucleate microtubules significantly earlier than the new SPB because recruitment of Spc72 – a core component of the SPB and a receptor for γ-Tubulin – to the new SPB occurs with a significant delay. Abolishing this difference by forcing simultaneous nucleation of astral microtubule from both the old and the new SPB causes randomization of SPB segregation [68]. This suggests that SPB segregation can result from structural asymmetries in the SPBs imposed by the SPB replication cycle. However, additional complexities are likely to exist. Using recombinase-dependent exchange of fluorescent tags fused to Spc72 to specifically label old and new SPBs allowed screening for genes involved in directional SPB segregation [69]. This approach revealed that Nud1/centriolin, a core structural component of the SPB, together with components of the mitotic exit network – a conserved signaling cascade controlling key events of exit from mitosis and cytokinesis – are required to specify the fate of the SPB [69]. Without a fully functioning mitotic exit network Kar9 does not preferentially recognize the old SPB and the older SPB is inherited randomly [69].

### 1.6 Retaining the ability to rapidly produce a primary cilium

Another structural difference between centrioles in vertebrate cells is linked to the fact that mother centrioles produce the primary cilium. The primary cilium is generated as mother centrioles mature into a basal body that is anchored at the membrane [70], [71]. In the case of radial glia, the non-random segregation of centrosomes could thus be linked to the fact that these cells are ciliated. Contrary to observations in other cell types, the primary cilium is not completely disassembled when absorbed prior to cell division in these cells. Remnants of it stay attached to the mother centrosome during mitosis and co-segregate to the daughter cell that retains stem cell characteristics [72]. Intriguingly, observations made in mouse fibroblasts already suggested that inheriting the older centrosome results in an asymmetric outcome for the timing of primary cilium production. Both fibroblast daughter cells can build a primary cilium, but the daughter cell inheriting the older centriole produces a primary cilium first. This asynchrony results in a differential response to Sonic hedgehog signaling [73]. Similarly, an asymmetry in the ability to form a cilium between progenitor cell daughters could lead to differences in their ability to respond to proliferative signals [72]. Hence inheriting the ability to rapidly produce a primary cilium by asymmetrically receiving mother centrioles might support maintenance of radial glial fate. Indeed, depletion of the mother centriole marker Ninein by RNAi led to a reduction in the number of progenitor cells, suggesting that losing mother centrosome specific markers from the centriole impacts on cell fate maintenance [54]. However, depletion of Ninein affects formation of the primary cilium in retinal pigment epithelial cells [74] opening the possibility that loss of radial glia cells induced by Ninein knockdown may not solely be attributable to loss of mother centriole traits, but could also be due to loss of cilium-mediated signal transduction. Thus, direct evidence for non-random centrosome segregation and progenitor cell fate is still missing. It will be important to dissect the role of the primary cilium in ciliated progenitor cell divisions to resolve this issue.

### 1.7 Molecules involved in centriole segregation in Drosophila neuroblasts

In *Drosophila* neuroblasts differences between centrosomes exist in interphase. One centriole nucleates an aster and is stably bound to the cell cortex, while the other does not nucleate microtubules and moves freely through the cytoplasm [60], [75]. Progress was made recently shedding light on the molecular details of this process. Centrobin (CNB), a protein specific for daughter centrioles that was first identified in mammalian cells is required for centriole duplication [76] and localizes to the daughter centriole in *Drosophila* [57], actively nucleating microtubules and cortex bound. In interphase neuroblasts, CNB is required to recruit the machinery that nucleates microtubules. Loss of CNB abolishes the ability of daughter centrioles to nucleate microtubules causing both centrioles to move apparently in a random manner within the cytoplasm. Loss of CNB also randomizes the centriole segregation pattern. [77]. Conversely, forcing recruitment of CNB to both centrioles leads to microtubule nucleation from both centrioles generating two cortex-bound asters close to each other [77]. In both cases total number of centrioles per cell is normal, but at least in the case of CNB loss, the stereotype inheritance of the daughter centriole by the neuroblast is lost, which is likely to happen when CNB is forced to both centrioles in these cells as well.

Recently Pericentrin like protein (PLP) was discovered as an additional player in regulating microtubule nucleation in interphase neuroblasts. PLP localizes to both centrioles, but higher levels accumulate on the mother centriole [78]. Loss of PLP causes activation of microtubule nucleation at both centrioles suggesting that PLP is normally involved in suppressing microtubule nucleation at the mother centriole [78]. Unlike loss of CNB, loss of PLP also compromises centrosome segregation, but leads to abnormal centrosome numbers per cell [78]. CNB and PLP are thus components that regulate microtubule nucleation and affect the stereotype segregation of centrioles.

### 1.8 Centrosomes and selective DNA strand segregation

Almost 40 years ago, the immortal strand hypothesis was proposed by John Cairns. It states that in order to protect themselves against mutation due to errors introduced by DNA replication, stem cells retain the original DNA template strand [79]. This hypothesis has been revised [80], [81] that stem cells might still control DNA strand segregation, but do so to differentially segregate epigenetic information. One major caveat is that molecular mechanisms that enable execution of this task are largely unknown [80]. The finding that labeling centrosomes in *Drosophila* male germ line stem cells within a short time window during embryogenesis was sufficient to generate label-carrying centrosomes many cell generations later in the adult, demonstrated the permanent presence of the same centrosome within male germ line stem cells [53]. Such an ‘immortal centrosome’ could be an element that provides continuity in controlling DNA strand segregation [82].

There is still no evidence of immortal DNA strands in the Drosophila male germ line [83], [84], [85]. Yet the finding that male germ line stem cells retain certain histones during asymmetric division [86] indicates that these cells might differentially transmit epigenetic information. In line with this idea, using chromosome oriented fluorescent *in situ* hybridization [87] non-random sister chromatid segregation of only the sex chromosomes was reported to occur in these cells [85]. The SUN-KASH domain containing proteins connect cytoplasmic elements of the cytoskeleton with the nuclear lamina and chromosomes [88]. This machinery might control non-random sister chromatid segregation since interfering with the centrosome or components of the SUN-KASH machinery randomized chromatid segregation [85]. Nonetheless, how individual DNA strands are recognized remains completely unclear, as does the role played by the mother centrosome in this process. Furthermore, randomizing DNA strand segregation by impaired centrosome function, did not immediately affect germ line stem cell fate or number [85], leaving the functional relevance of this phenomenon unclear.

### 1.9 Cell intrinsic memory of spindle orientation

Neuroblasts are special because they are the only somatic cells in Drosophila with a centrosome actively nucleating microtubules during interphase [59], [60], [89]. It is also notable that in these cells the daughter centriole recruits the machinery to nucleate microtubules in interphase [77], a feature typically performed by the mother centriole in other systems [90]. In interphase Drosophila neuroblasts, the daughter centriole organizes a microtubule aster that keeps an invariant position at the cortex, which will become the apical pole in the next mitosis and hence remains in the neuroblast. Therefore the interphase microtubule aster is located opposite from the position where daughter cells are born [60], [75], [91]. Why daughter cells cluster remains unclear, but in the *Drosophila* embryo, mechanisms exist to correct errors in the orientation of neuroblasts division that involve signaling from neighboring glial cells [92], suggesting that daughter cell clustering is of critical importance during central nervous system development in *Drosophila*. In larval neuroblasts, the position of the microtubule aster at the apical cell pole opposite to the daughter cell cluster suggested that it might play a role in transmitting cell division orientation information from one division to the next. Consistent with this idea, transiently disrupting microtubules, which leads to loss of asters and the anchoring of centrioles to the cortex, resets the orientation of divisions by establishing an ectopic microtubule aster that serves as a predictor of the new axis of division after restoring microtubule dynamics [93].

Mutants such as *mud* induce an increase in the number of symmetric divisions of neuroblasts thus interrupting the normal pattern of asymmetric divisions [13], [14], [94]. Subsequent asymmetric divisions of the resulting *mud* mutant neuroblast siblings respect the orientation of the preceding symmetric cell division and daughter cells are born into the space between the sibling neuroblasts pair [95]. This means that in this case the orientation of the preceding divisions is maintained. These data suggest that neuroblasts can ‘read’ or remember the orientation of their last division. The responsible mechanism is not clear. However, the memory of division orientation also functions robustly when the interphase aster is composed of two centrosomes. On the other hand, it is prone to errors when centrosome function is impaired or when microtubule dynamics are disrupted [77], [78], [93]. This suggests that it is important for neuroblasts to have a functional microtubule network in interphase for the cell polarity memory to work, but why the system requires the daughter centrosome remains unknown.

### 1.10 Regulation of centrosome segregation by signaling pathways

An important question that remains is whether cell extrinsic input contributes to bias in centrosome segregation. Orientation of cell division is known to be regulated by a number of signaling events between cells [96]. The Wnt/planar cell polarity (PCP) pathway can regulate spindle orientation [97]. Remarkably Wnt signaling seems to be able to bias centrosome segregation. When exposed to a localized source of Wnt3a signal, embryonic stem cells in culture can be triggered to show biased centrosome segregation taking the older centrosome to the cell closer to the source of Wnt3a. The cell retaining this centrosome was also seen retained pluripotency markers [98]. However, the molecular details of how exposure to Wnt regulates the orientation of mitotic spindles are not well understood. In *Drosophila* and zebrafish the transmembrane receptor Frizzled and its effector Dishevelled (Dsh) are involved [99]. They can interact with Mud/Numa linking Wnt signaling to the spindle orientation machinery [99]. That means it is possible that a similar signaling event also provides cues for the attraction of one spindle pole, the one containing stronger Ninein signal, a marker for the mother centriole, in embryonic stem cells [98].

We do not understand the signaling that governs the selection of one spindle pole over the other, but details about how downstream targets of Wnt signaling could contribute to the orientation of mitosis are emerging. Wnt-dependent spindle orientation, recently identified in zebrafish dorsal epiblast cells, showed involvement of the anthrax toxin receptor 2a [100]. Wnt polarizes the activity of this receptor. In cooperation with RhoA it activates the formin zDia2 to locally generate actin filaments to help orient the spindle [100]. The precise role of actin cables in spindle positioning remains to be determined.

In Drosophila S2 cells, experimentally forcing the localization of Dsh to restricted cortical regions causes recruitment of the actin binding protein Canoe/Afadin locally activating Rho signaling. Dia then functions as an effector of Rho activation inducing F-actin enrichment at sites of cortical Dsh [101].

Interestingly, during *Drosophila* neuroblast asymmetric divisions Canoe is involved in spindle orientation [102] by playing a role in recruiting Mud [103]. These results from zebrafish and *Drosophila* indicate that actin–dependent processes might influence spindle orientation similar to the situation in budding yeast. In yeast, actin cables serve to guide astral microtubules to position the spindle during mitosis [104]. Alternatively, the interaction of Pins/Canoe could be a way to stabilize the cortical position of Galphai/Pins/LGN/Mud/Numa complexes [105]. It will be important to test whether the actin–myosin network is involved in this process in cells where non-random centrosome segregation occurs.

Another signaling pathway that was recently implicated in asymmetric centrosome behavior is the Notch signaling pathway. In cells of the peripheral nervous system of *Drosophila*, asymmetries in centrosome behavior correlate with differences in centriole migration. During cytokinesis of the sensory organ precursor cell the anterior and posterior centrosome differed in the time required for their movement to the apical pole. Notably, this differential movement was delayed in mutants of Numb, a regulator of the Notch pathway, and accelerated when Numb was overexpressed [106] suggesting that Numb regulates differential centrosome behavior in this cell type. Consistent with this idea, Notch may also function in regulating spindle orientation in the mammary epithelium. Treating young mice with γ-secretase inhibitor to block Notch signaling was reported to result in measurable differences in the orientation of mitosis in cells within the terminal end buds [107]. Hence, in addition to the well-known link between asymmetric cell division and the control of Notch pathway activity, Notch signaling might play also a more direct role as a regulator of centrosome and spindle behavior.

## 2 Conclusion

Many potential mechanisms have emerged that contribute to the phenomenon of non-random segregation of centrosomes. These include differences in their structure and molecular composition, and in their ability to respond to specific signals. Observations from yeast show that even if structural differences can suffice to ensure asymmetric SPB segregation [68], additional layers of regulation that involve signaling cascades can impact on SPB behavior [69]. Similar to the situation in yeast, centrosome segregation seems to be controlled in a sophisticated manner in *Drosophila* neuroblasts since: (1) pericentriolar material is actively shed from the mother centriole at the end of mitosis and accumulates on the daughter centriole [56], [59]; (2) stable microtubule nucleation by the daughter centriole requires the action of Pins, a protein that has thus far been shown to only localize to the apical cortex in mitosis [59], [77]. Thus, in *Drosophila* neuroblasts and yeast signals that control biased centrosome/SPB segregation cannot solely be explained by structural differences in centriole maturation.

It is also still unknown whether the loss of a primary cilium from progenitor cells affects their fate. To this end, it will be important to determine if depleting specific genes, such as ODF2, which renders mother and daughter centrioles indistinguishable at the ultra-structural level and prevents primary cilium formation without impinging on the cell cycle [108], affects progenitor fate.

Importantly, a clear-cut connection between directed centrosome segregation and cell fate generation has not been demonstrated in any of the systems that exhibit non-random centrosome segregation. To this end, it will be most informative to investigate now whether asymmetric centrosome segregation is (i) a general feature of stem cell division, (ii) occurs only during asymmetric division or can also be observed in symmetric divisions and (iii) occurs in cells in which non-random segregation of DNA strands occurs. It should now be possible to measure this in muscle satellite cells, crypt stem cells and intestinal stem cells in *Drosophila* [24], [81], [109], [110], [111]. The most important point to resolve will be to establish how non-random centrosome segregation and cell fate are related to test the beautiful hypothesis that inheriting one type of centrosomes ensures the continuity of cell fate between different generations.
